# Factors influencing neurocognitive function in patients with neuroepithelial tumors

**DOI:** 10.1038/s41598-017-17833-w

**Published:** 2017-12-19

**Authors:** Jens Gempt, Nicole Lange, Stefanie Bette, Sarah Charlotte Foreman, Jasmin Hernandez Cammardella, Jennifer Albertshauser, Corinna Gradtke, Niels Buchmann, Yu-Mi Ryang, Friederike Schmidt-Graf, Bernhard Meyer, Florian Ringel

**Affiliations:** 1 Neurochirurgische Klinik und Poliklinik, Klinikum rechts der Isar, Technische Universität München, Ismaninger Str. 22, 81675 München, Germany; 2 Abteilung für Neuroradiologie, Klinikum rechts der Isar, Technische Universität München, Ismaninger Str. 22, 81675 München, Germany; 3 Neurologische Klinik und Poliklinik, Klinikum rechts der Isar, Technische Universität München, Ismaninger Str. 22, 81675 München, Germany

## Abstract

Though cognitive function is proven to be an independent predictor of survival in patients with intrinsic brain tumors, cognitive functions are still rarely considered. Aim of this study was to assess neurocognitive function and to identify risk factors for neurocognitive deficits. 103 patients with primary neuroepithelial tumors who received tumor resections or biopsies were included in this prospective study. The following data was acquired: mini-mental state examination, preoperative tumor volume, WHO grade, tumor entity and location, and the Karnofsky performance status scale. Furthermore, patients participated in extensive neuropsychological testing of attentional, memory and executive functions. General factors like age, clinical status, WHO grade, tumor volume and tumor location correlated with patients’ neurocognitive functions. Affection of the parietal lobe resulted in significant impairment of attention and memory functions. Frontal lobe involvement significantly affected patients’ abilities in planning complex actions and novel problem solving. Patients with temporal lesions were more likely to have impaired memory and executive functions. Comparing results among neuroepithelial tumor patients enables the identification of risk factors for cognitive impairment. General parameters such as age, KPS score, tumor size, and WHO grade are apart from the respective tumor location of high importance for neurocognitive function.

## Introduction

Neurocognitive impairment is commonly observed in patients with primary brain tumors^1^. Though it has been shown that cognitive function is an independent predictor of survival in patients with primary brain tumors, cognitive functions are still rarely considered^2,3^. Most oncological studies dealing with brain tumor patients assess the Karnofsky Performance Status score (KPS), or evaluate cognitive function with simple tools like the MMSE^4,5^.

Generally functional assessment of the afflicted organ is crucial for all oncological diseases. In case of pulmonary tumors spirometry and blood-gas analysis are feasible methods to evaluate lung function. For hepatic cancer different laboratory diagnostics of liver synthesis can help to estimate the residual function. As brain function is complex and multifaceted, a pure structural assessment including different imaging modalities, laboratory diagnostics, and basic neurological examinations, will only allow a limited deduction of higher neurocognitive functions.

To date, there are several studies illuminating different factors influencing cognitive dysfunction in patients with brain tumors. Though these are predominantly studies with limited patient numbers and preselected lesion location^6,7^.

In more recent studies lesion momentum is discussed as an important risk factor for neurocognitive deficits in patients with intrinsic brain tumors^8–10^. According to the principles of neuroplasticity this means that faster growing lesions permitting little time for the brain to adapt might result in more distinct neurocognitive deficits even in case of smaller absolute tumor volume.

As a variety of methods are used by different studies to assess neurocognition in brain tumor patients, a direct comparison of results is difficult^11^.

In this study we systematically assessed neurocognitive functions in patients with primary neuroepithelial brain tumors. Our primary objective was to identify risk factors for neurocognitive deficits in a larger patient cohort and thus to facilitate pre-treatment risk assessment.

## Material and Methods

### Patients

In this study adult patients with neuroepithelial tumors that received tumor resections or biopsies were invited to participate. The study was approved by the local ethics committee (Ethikkommission der Technischen Universität München) and performed in accordance with the ethical standards of the 1964 Declaration of Helsinki and its later amendments (Clinical Trial Registration Number: 2840/10). All patients signed an informed consent form. Data was acquired in a prospective clinical setting. Patients with severe psychiatric disorders, that did not sign the informed consent form, with urgent need for surgery, and contraindications for MRI examinations were excluded. In addition personality traits were evaluated in a subgroup of patients. These results were reported elsewhere.

Patient and disease characteristics were recorded. Tumor location, histopathological findings, and the Karnofsky Performance Status score (KPS) were noted. The pre-treatment tumor volume was assessed by manual segmentation of T2-weighted fluid attenuated inversion recovery (FLAIR) MRI sequences and T1-weighted contrast enhanced sequences using IPlannet (Iplan 3.0, Brainlab AG, Feldkirchen, Germany). All patients underwent preoperative cognitive evaluation with standardized neuropsychological tests according to the following protocol.

### Outcome measures

#### Basic test battery

The basic test battery included the mini-mental state examination (MMSE). Patients that scored 19 points or more in the MMSE were subjected to the extended neuropsychological test battery. For patients with an MMSE score below 19 points, testing was completed with the basic test battery only.

#### Mini-Mental State Examination

The MMSE is a well-established cognitive screening instrument. It is used to measure cognitive impairment by examining functions such as registration, attention, calculation, language, recall, orientation, and ability to follow simple commands. The maximum score is 30 points. A score of 24 points or more is defined as normal cognition. A score below 23 points is defined as cognitive impairment (19–23 points: mild impairment; 10–18 points: moderate impairment; <9 points: severe impairment)^12^.

### Extended test battery

#### Attention

The subtest *alertness* of the computer-based *test battery of attentional performance* (TAP)^13^ was used to measure intrinsic alertness and reaction time. In this subtest, visual stimuli are displayed on a computer screen, and subjects are required to respond to the appearance of each stimulus as fast as possible by a pressing a button. The subtest consists of two testing conditions. In the first condition, visual stimuli are displayed exclusively. In the second condition, visual stimuli are preceded by an auditory cue. Reaction times in both conditions were used as dependent variables in the present study.

The subtest for *divided attention* was used to measure patients’ ability to simultaneously process two different kinds of stimulus presentations (visual and auditory). The visual task consists of recognizing a square of four crosses in a constantly changing pattern of crosses on a computer screen (visual task). At the same time, the subjects are required to register two immediately successive identical tones (deep-deep or high-high) from a sequence of high and low tones (auditory task). For both tasks, subjects are instructed to respond by pressing a button as quickly as possible. The response times, the lack of response to both stimuli and the reactions to which no stimulus can be attributed are measured.

The *d2 test*^14^ is one of the most frequently used psychological tests worldwide to assess patients’ attention and concentration performance. In this paper – pencil task, subjects are required to identify and cross out all d’s with exactly two strokes from a series of d and p’s with a different number of strokes above or below. Speed (amount of time needed to complete the task) and diligence (number of mistakes made) are measured.

The *Trail Making Test, version A* (TMT-A)^15^ is one of the two parts of the Trail Making Test. It is a popular and widespread neuropsychological test, which helps to evaluate patients’ visual search abilities, scanning and processing speed, intellectual flexibility, as well as executive functions. In this test part, patients are required to join numbers (1 to 25) written on a piece of paper in the right order as quickly as possible by connecting them with a pen. The time required to finish the task correctly is measured.

#### Memory

The test battery of the *Wechsler Memory Scale revised (WMS-r)*^16^ consists of a total of thirteen subtests, of which the subtests *block- and digit span* were used in this study. This subtest can be used to evaluate verbal and figurative short-term memory by determining the extent to which verbal and visual material can be reproduced directly and with short delay. For the *digit-span* part a row of up to nine digits is read to the subjects, which then has to be recalled first in the same order, then in reverse order. The number of mistakes is noted. For the visual memory span part (also referred to as the *block span)*, a group of randomly arranged wooden blocks on a board is first touched by the examiner in a certain sequence (again two to nine episodes). Then subjects are required to repeat the sequence first in the same order, then reversed. Again, the number of mistakes is noted.

The *verbal learning and memory test* (VLMT)^17^ is a serial list-learning test, used to assess declarative-episodic memory function. The VLMT includes a learning list and an interference list with fifteen semantically independent words each (e.g. house, sun, boat). After five passages of reading out the learning list to the patients (D1–5) with subsequent interrogation of the memorized words, the interference list (DI) is read out once. Patients are then requested to repeat the memorized words from the learning list immediately (D6) and again after 30 minutes (D7).

The *Rey Osterrieth complex figure test* (ROCF)^18^, is a widespread neuropsychological test, used to examine spatial visual constructive capacity as well as visual memory performance. The ROCF consists of a pattern of different geometrical elements, which can be decomposed into eighteen units. Patients are first shown the figure, then instructed to draw this figure from memory. Position, accuracy and organization of the elements are evaluated. A maximum of two points can be obtained for each of the eighteen units.

#### Executive functions

*Version B* of the *Trail Making Test* (TMT-B)^15^ was used to assess patients’ ability to switch tasks. In the test patients were asked to alternately connect letters and numbers in the right order (e.g. 1-A-2-B-3-C). The time required for this task was measured.

The *Stroop color-word test*^19^ is a sensomotoric performance test used to evaluate basic cognitive functions of reading, naming, and the ability of selective attention. The three categories color-word-reading, color-naming and interference are aligned to assess patients’ ability in processing information in the optical-verbal function area. In the first part, the patient is instructed to read the words “red”, “yellow”, “green” and “blue” printed in black color as quickly as possible. In the second part the patient is required to name the colors of different colored strokes (again “red”, “yellow”, “green” and “blue”). In the last part (interference attempt), the test panel consists of color words which are written in a different color from the word itself (e.g. the color word “blue” is written in the color red – color-word-incongruence). In addition to the time required for each task, the number of corrected and uncorrected errors are recorded in order to assess patients’ transfer performance.

The *Regensburg word fluency test* (RWT)^20^ was developed for German-speaking areas in order to examine lexical fluency and semantic capacities. The patient is asked to name as many words as possible in one minute beginning with a specific letter (lexical fluency). Numbers, names, words of the same word stem, or repeating the same word are considered as errors. Subsequently, the ability to change categories (e.g. H-T) is examined to determine the ability of reactive cognitive flexibility. To assess semantic capacities, the patient is then requested to name as many words as possible of one category (in our example: animals). The number of correct words are noted. It should be noted that impaired language performance may influence test results, mimicking lower executive functions.

An overview of the different tasks per cognitive domain is given in Table 1.

**Table 1:** overview of the tasks per cognitive domain.

cognitive domain	test	subgroups
Attention	TAP	TAP Alertness W/O sound
		TAP Alertness W_sound
		TAP Alertness phasic
		TAP divided attention visual
		TAP divided attention auditive
		TAP divided attention failure
		TAP divided attention selected
	D2	D2 GZ
		D2 GZ-F
		D2 F%
	TMT-A	TMT A
Memory	VLMT	VLMT Dg1
		VLMT Dg5
		VLMT Dg1_5
		VLMT Dg6
		VLMT Dg7
		VLMT Dg5_6
		VLMT Dg5_7
	WMS	WMS ms v
		WMS wm v
		WMS ms nv
		WMS wm nv
	ROCF	ROCF Copy
		ROCF Delay
Executive functions	TMT B	TMT B
	Stroop’s	Stroop’s word reading
		Stroop’s line naming
		Stroop’s interference
		Stroop’s failure
	RWT	RWT formallexical
		RWT semantic
		RWT turning f/l
		RWT turning semantic

#### Statistical data analysis

Statistical analyses, including descriptive data analyses, were performed using IBM SPSS Statistics version 22.0 (IBM Corporation, New York). Data was analyzed regarding normal distribution (histograms, QQ-plots, Kolmogorov-Smirnov- and Shapiro-Wilk-test). Normally distributed data are shown as mean and standard deviation, correlation analysis was conducted with the Pearson correlation coefficient, comparisons of mean values were conducted with t-tests. Non-normally distributed data are shown as median and interquartile range (IQR) and were analyzed with Spearman’s rank correlation coefficient, Whitney-U- or Kruskal-Wallis-tests. Furthermore, Chi-square-Automatic-Interaction-Detector (CHAID) was used to analyze the MMSE results. CHAID is a decision tree based on adjusted significance testing specifications. We chose a maximum tree depth of 3, a minimum number of cases in parent-nodes of 20 and of 10 in child-nodes.

We conducted it to identify significant factors that influence basic neurocognitive function according to MMSE. For all analyses, a difference with an error probability of less than 0.05 was considered to be statistically significant. Considering that this is an exploratory study, no further correction for multiple testing was performed.

For further detailed analysis according to the influence of tumor location in combination with further parameters, a generalized linear model (multiple linear regression with Wald-Chi-Square) was used.

## Results

### Clinical data

Of 197 patients that were invited to participate in this study, 112 patients agreed to participate. Nine patients were excluded due to histopathological results not compatible with the histopathological criteria of intrinsic brain tumors. Thus a total of 103 patients were included. The mean patient age was 51 years (range 18 to 84 years, 49 f, 54 m).

Tumor grades and entities were: WHO IV = 51 (Glioblastomas (n = 49), Gliosarcoma (n = 1), Medulloblastoma (n = 1)); WHO III = 13 (Anaplastic Astrocytomas (n = 8), Anaplastic Oligoastrocytomas (n = 5)); WHO II = 18 (Diffuse Astrocytomas (n = 11), Oligodendrogliomas (n = 3), Oligoastrocytomas (n = 2), Neurocytomas (n = 2)); WHO I = 22 (Pineocytomas (n = 11), Pilocytic Astrocytomas (n = 4), Gangliogliomas (n = 3), Subependymomas (n = 2), Dysembryoblastic neuroepithelial tumors (n = 2)).

The mean tumor volume as assessed by MRI was 16.98 cm^3^ in T1-weighted post contrast sequences (0.00 to 165.32 cm^3^, SD 27.44) and 42.88 cm^3^ in FLAIR-weighted sequences (0.00 to 231.97 cm^3^, SD 51.49).

26 tumors were located in the frontal lobe, 4 in the parietal lobe, 17 in the temporal lobe, 1 in the occipital lobe, and 37 tumors were located in multiple lobes.

The median preoperative Karnofsky Performance Status score (KPS) was 90%. Higher WHO tumor grades were associated with lower initial KPS scores (WHO I = 89%, WHO II = 86%, WHO III = 79%, WHO IV = 76%). An overview of the clinical characteristics of the patient collective is provided in Table 2.

**Table 2:** Patient demographic and baseline characteristics.

Age (years; mean, range)	51 (66)
**sex (N**=**)**	
female	49
male	54
**Tumor volume (mean, SD)**	
T2 flair (cm³)	42.88 (51.49)
T1 contrast enhanced (cm³)	16.98 (27.44)
**WHO Grade (N=)**	
I	22
Pineozytoma	11
Pilocytic Astrocytoma	4
Ganglioglioma	3
Subependymoma	2
Desembryoblastic neuroepithelial tumor	2
II	18
Diffuse Astrocytoma	11
Oligodendroglioma	3
Oligoastrocytoma	2
Neurocytoma	2
III	12
Anaplastic Astrocytoma	8
Anaplastic Oligoastrocytoma	4
IV	51
Glioblastoma	49
Gliosarcoma	1
Medulloblastoma	1
**Tumor location (N=)**	
frontal lobe	26
parietal lobe	4
temporal lobe	17
occipital lobe	1
other	18
multiple lobes	37
**Education (graduation, N=)**	
unknown	9
none	1
main school (Hauptschule)	35
secondary school (mittlere Reife)	25
A-Level (Abitur)	33

### Cognitive assessment

#### Basic test battery

All patients included in the study underwent MMSEs, which represented the basic test battery of this study. The number of points scored in the MMSE showed a strong inverse correlation with the WHO grading of the tumor (WHO I = 28.1 points, WHO II = 28.3, WHO III = 25.6, WHO IV = 24.5; k = −0.453; p = 0.000). This was also the case for the parameter patient age (k = −0.613, p = 0.000). Regarding tumor location, patients with tumors located in the parietal lobe had the lowest MMSE scores (mean 25.75 points, SD 2.630), followed by patients with multiple affected lobes.

**Figure 1:**
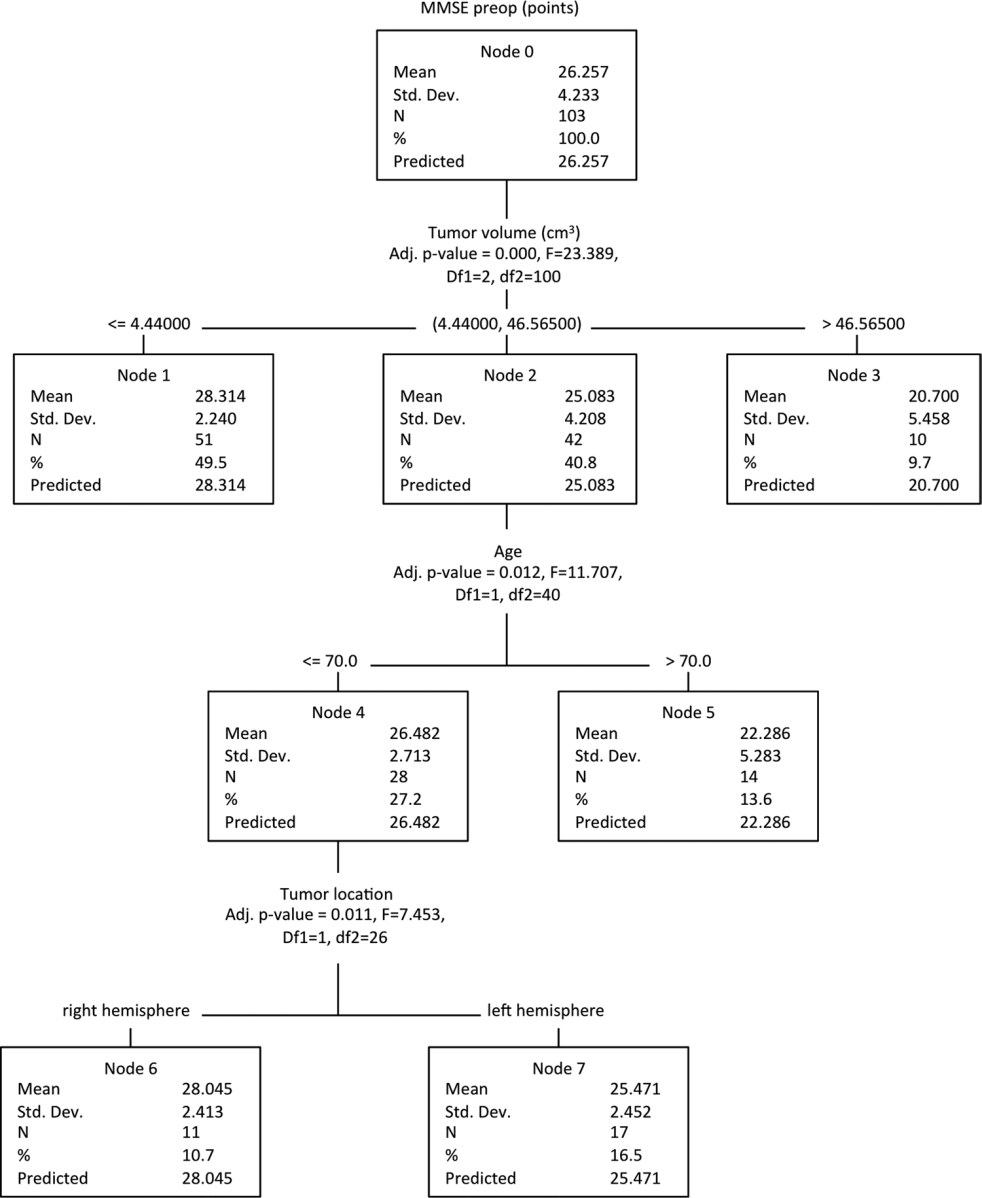
Classification tree analysis of the parameters influencing MMSE scores, revealing tumor volume, age and involvement of the left hemisphere as significant.

Furthermore, a CHAID-tree-analysis was conducted (Fig. 1). The maximum tree depth was set to 3, the minimum cases in parent node were 20, and 10 in child nodes. According to the CHAID-tree-analysis, factors significantly associated with lower MMSE scores were tumor volume, patient age, and tumor location in the dominant hemisphere.

Tumor volume was selected as node 0 with a p-value of 0.000, dividing patients into the nodes: tumor volume <4.44 cm^3^ (node 1) with a mean MMSE score of 28.3, tumor volume >4.44 cm^3^ and <46.57 cm^3^ (node 2) with a mean MMSE score of 25.1, and tumor volume >46.57 cm^3^ (node 3) with a mean MMSE score of 20.7.

From node 2 onwards patients were further subdivided into groups by the parameter patient age (p = 0.012): <=70 years (node 4) and >70 years (node 5) with MMSE scores of 26.5 and 22.3, respectively. From node 4 onwards, patients were subdivided into groups by the parameter tumor location (location in the left hemisphere, p = 0.011) with MMSE scores of 25.5 (left hemisphere affected) and 28.0.

#### Attention

For further detailed analysis of the attention category tests a generalized linear model (multiple linear regression with Wald-Chi-Square) was used. The following parameters were included: patient age, affected lobe, tumor volume in FLAIR-weighted sequences, KPS score, and WHO grade (Table 3).

**Table 3:** Results of the attention category tests and subtest of the multiple linear regression (with Wald-Chi-Square) analysis.

		age	tumor volume	WHO-grade	KPS	frontal lobe		temporal lobe		parietal lobe	
						right	left	right	left	right	left
TAP	TAP neglect MD_R	0.000	n.s.	0.000	n.s.	n.s.	0.02	n.s.	n.s.	n.s.	n.s.
	TAP neglect MD_L	0.000	n.s.	0.003	n.s.	n.s.	n.s.	n.s.	n.s.	n.s.	n.s.
	TAP neglect skip_R	0.008	n.s.	n.s.	0.043	n.s.	0.035	n.s.	n.s.	n.s.	n.s.
	TAP neglect skip_L	0.005	0.001	n.s.	n.s.	n.s.	0.014	n.s.	n.s.	n.s.	n.s.
	TAP neglect skip_C	0.008	n.s.	n.s.	n.s.	n.s.	0.050	n.s.	n.s.	n.s.	n.s.
	TAP Alertness W/O sound	n.s.	n.s.	0.001	0.012	n.s.	0.014	n.s.	n.s.	n.s.	n.s.
	TAP Alertness W_sound	0.019	n.s.	0.005	0.006	n.s.	n.s.	n.s.	n.s.	n.s.	n.s.
	TAP Alertness phasic	n.s.	0.014	0.033	n.s.	n.s.	0.001	0.000	n.s.	n.s.	n.s.
	TAP divided attention visual	0.001	n.s.	0.038	0.000	n.s.	0.004	n.s.	n.s.	n.s.	n.s.
	TAP divided attention auditive	n.s.	n.s.	n.s.	n.s.	n.s.	n.s.	n.s.	n.s.	n.s.	n.s.
	TAP divided attention failure	0.009	0.002	0.042	n.s.	n.s.	n.s.	n.s.	n.s.	n.s.	n.s.
	TAP divided attention selected	0.002	n.s.	0.001	n.s.	n.s.	n.s.	n.s.	n.s.	n.s.	n.s.
D2	D2 GZ	0.001	n.s.	0.000	n.s.	n.s.	n.s.	n.s.	n.s.	0.032	n.s.
	D2 GZ-F	0.000	n.s.	0.001	n.s.	n.s.	n.s.	n.s.	n.s.	n.s.	n.s.
	D2 F%	0.02	0.05	n.s.	n.s.	n.s.	n.s.	n.s.	n.s.	0.003	n.s.
TMT A	TMT A	n.s.	0.025	0.01	0.002	0.021	0.027	0.007	n.s.	0.003	0.001

Parameters included were: age, affected lobe, tumor volume in FLAIR-weighted sequences, KPS score, and WHO grade. Significant p-values are listed. For a decline in neurocognitive function the p-values are listed black, for improved neurocognitive function p-values are white.

Higher age was significantly associated with lower scores in nearly all subtests for attention (significance level <0.01 in 10/15 subtests and <0.02 in 2/15 subtests). The parameter tumor volume was significantly associated with lower cognitive function with a p-value of <0.05 in 3/15, and p < 0.01 in 2/15 subtests. WHO grade was highly significant in most of the subtests (p < 0.01 in 8/15 subtests and p < 0.05 in 3/15 subtests). Higher WHO grade was associated with lower test scores. Clinical status evaluated by KPS scores was significantly associated with 5/15 of the attention tests at a significance level of p < 0.01 in 3/15 subtests and p < 0.05 in 2/15 subtests. A higher KPS score was associated with higher cognitive function.

Tumor location in the left frontal lobe was associated with lower test scores in the attention test battery (p < 0.01 in 2/15 subtests and p < 0.05 in 6/15 subtests). Tumor location in the right parietal lobe was associated with lower D2 and TMT A test scores (p < 0.01 in 2 subtest and p < 0.05 in 1 subtest). Tumor location in the right temporal lobe was significant with a p = 0.000 for lower scores in the TAP phasic alertness test.

#### Memory

Patients with tumors located in the dominant hemisphere had significantly lower tests scores in all subtests of the VLMT and in 2/4 subtests of the WMS compared to patients with tumors located in the non-dominant hemisphere (Mann-Whitney-U-test, p < 0.05).

For further analysis the following parameters were included in the generalized linear model (multiple linear regression with Wald-Chi-Square, Table 4): patient age, affected lobe, tumor volume in FLAIR-weighted sequences, KPS score, and WHO grade.

**Table 4:** Results of the memory category tests and subtest of the multiple linear regression (with Wald-Chi-Square) analysis.

		age	tumor volume	WHO-grade	KPS	frontal lobe		temporal lobe		parietal lobe	
						right	left	right	left	right	left
VLMT	VLMT Dg1	0.001	n.s.	0.021	n.s.	0.037	n.s.	n.s.	n.s.	n.s.	n.s.
	VLMT Dg5	0.000	0.008	n.s.	n.s.	n.s.	0.002	n.s.	n.s.	n.s.	n.s.
	VLMT Dg1_5	0.000	n.s.	n.s.	n.s.	0.021	0.014	n.s.	n.s.	n.s.	n.s.
	VLMT Dg6	0.000	0.023	0.015	n.s.	0.018	n.s.	0.001	n.s.	n.s.	n.s.
	VLMT Dg7	0.000	0.027	n.s.	n.s.	0.021	n.s.	0.030	n.s.	n.s.	n.s.
	VLMT Dg5_6	n.s.	n.s.	0.017	n.s.	n.s.	0.012	0.038	0.016	n.s.	n.s.
	VLMT Dg5_7	n.s.	n.s.	n.s.	n.s.	n.s.	n.s.	n.s.	n.s.	n.s.	n.s.
WMS	WMS ms v	n.s.	n.s.	n.s.	n.s.	0.026	n.s.	n.s.	n.s.	n.s.	0.005
	WMS wm v	n.s.	n.s.	0.014	n.s.	n.s.	n.s.	n.s.	n.s.	n.s.	0.009
	WMS ms nv	n.s.	n.s.	n.s.	n.s.	n.s.	n.s.	n.s.	n.s.	n.s.	n.s.
	WMS wm nv	0.002	n.s.	0.020	n.s.	n.s.	n.s.	n.s.	n.s.	n.s.	n.s.
ROCF	ROCF Copy	n.s.	0.012	n.s.	0.000	n.s.	n.s.	n.s.	n.s.	n.s.	n.s.
	ROCF Delay	0.005	n.s.	0.035	n.s.	0.006	n.s.	n.s.	n.s.	n.s.	n.s.

Parameters included were: age, affected lobe, tumor volume in FLAIR-weighted sequences, KPS score, and WHO grade. Significant p-values are listed. For a decline in neurocognitive function the p-values are listed black, for improved neurocognitive function p-values are white.

Memory function test scores were significantly lower in patients with higher age, higher WHO grade, lower KPS scores, and higher tumor volume. Age was significant in 7/13 subtests, with a significance level of p < 0.001. Tumor volume had a significant influence in 3/13 subtests with a significance level of p < 0.05 and p < 0.01 in 1/15 subtests. Clinical status evaluated by KPS scores was significant in the ROCF test (copy) with a significance level of p = 0.000. WHO grade was significant in 6/15 subtests with a significance level of p < 0.05.

Patients with lesions located in the right frontal lobe had significantly higher test scores in 6/13 subtests (p < 0.05 in 5 subtests and p < 0.01 in the ROCF Delay subtest) compared to patients with lesion located in the left frontal lobe.

Reduced cognitive memory functions were observed for patients with the following tumor locations: left parietal lobe (2/13 subtests; p < 0.01), right temporal lobe (3/13 subtests, p < 0.05 and 1/13 subtests, p < 0.01), and left temporal lobe (p < 0.01 in 1/13 subtests).

#### Executive functions

The generalized linear model (multiple linear regression with Wald-Chi-Square, Table 5) included the following parameters: patient age, WHO grade, affected lobe, tumor volume in FLAIR-weighted sequences, and KPS score.

**Table 5:** Results of the executive category tests and subtest of the multiple linear regression (with Wald-Chi-Square) analysis.

		age	tumor volume	WHO-grade	KPS	frontal lobe		temporal lobe		parietal lobe	
						right	left	right	left	right	left
TMT B	TMT B	0.006	n.s.	n.s.	0.003	n.s.	n.s.	n.s.	n.s.	n.s.	n.s.
Stroop’s	Stroop’s word reading	n.s.	n.s.	n.s.	0.000	n.s.	n.s.	n.s.	n.s.	n.s.	n.s.
	Stroop’s line naming	n.s.	0.000	n.s.	n.s.	n.s.	0.002	n.s.	n.s.	n.s.	n.s.
	Stroop’s interference	n.s.	0.000	n.s.	n.s.	n.s.	0.000	n.s.	n.s.	n.s.	n.s.
	Stroop’s failure	n.s.	n.s.	n.s.	n.s.	n.s.	n.s.	n.s.	n.s.	n.s.	n.s.
RWT	RWT formallexical	n.s.	n.s.	n.s.	0.005	n.s.	n.s.	n.s.	n.s.	n.s.	n.s.
	RWT semantic	n.s.	0.000	n.s.	0.045	n.s.	n.s.	n.s.	n.s.	n.s.	n.s.
	RWT turning f/l	n.s.	n.s.	n.s.	0.002	n.s.	n.s.	n.s.	n.s.	n.s.	n.s.
	RWT turning semantic	0.026	0.011	n.s.	n.s.	n.s.	0.036	n.s.	n.s.	n.s.	n.s.

Parameters included were: age, affected lobe, tumor volume in FLAIR-weighted sequences, KPS score, and WHO grade. Significant p-values are listed. For a decline in neurocognitive function the p-values are listed black, for improved neurocognitive function p-values are white.

Higher patient age was significantly associated with lower scores in 2/9 subtests for executive functions (p < 0.001 in 1/9 subtests and p < 0.05 in 1/9 subtests). Larger tumor volume was highly significantly associated with lower scores in 4/9 subtests (p = 0.000 in 3/9 and p = 0.011 in 1/9 subtests). Tumor location in the left frontal lobe was significantly associated with lower test results in 3/9 subtests (significance level p < 0.002 in 2/9 subtests and p = 0.036 in 1/9 subtests).

According to the model, cognitive function was significantly lower in patients with higher age, higher tumor volume, lower KPS scores, and with tumor location in the left frontal lobe.

**Figure 2:**
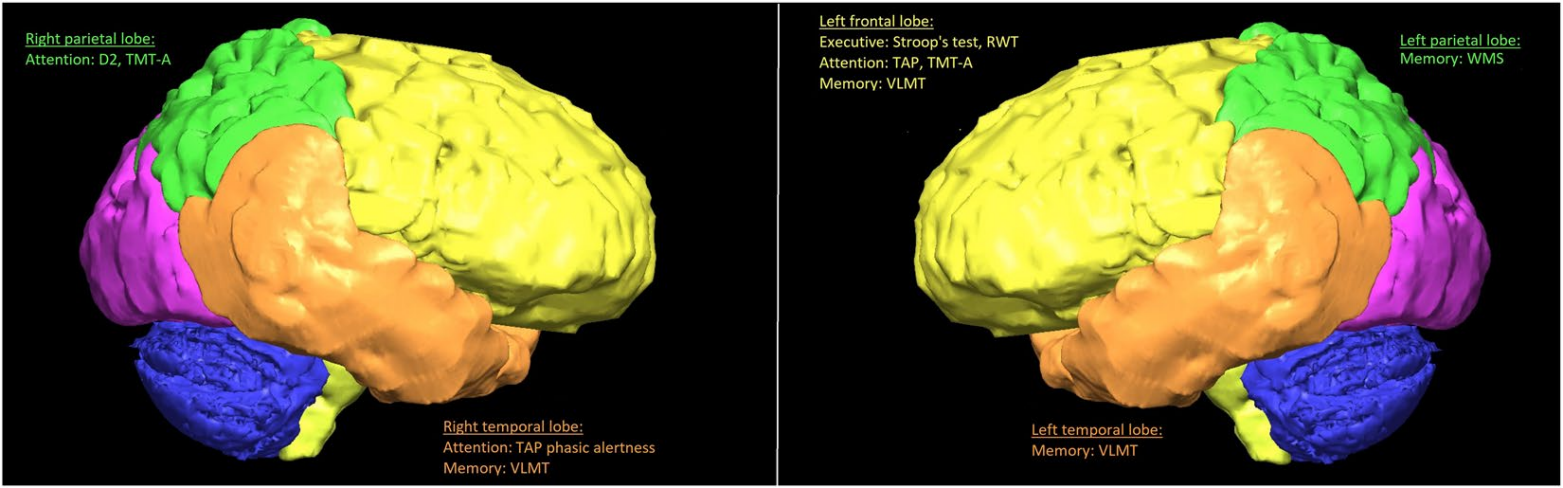
Significant differences in test results matched to the affected lobes – Affection of the frontal lobe leads to impairment of all three categories, lesions of the temporal lobe seem to reduce attention and memory functions, while parietal tumors affect patients’ memory and attention abilities.

Figure 2 demonstrates the affected test results according to lesion location.

## Discussion

In this study, factors that influence different types of cognitive dysfunction were identified by conducting complex neuropsychological tests for several different categories on 103 patients with primary brain tumors prior to surgery.

Test scores of patients with higher age, higher WHO grading, larger tumor volume, and lower KPS scores were significantly lower in multiple domains of our standardized neuropsychological testing protocol. Our series also showed that patients’ neurocognitive functions varied considerably depending on the lobe affected by the tumor. To our knowledge, prior studies have not analyzed the relationship between tumor location and neuropsychological test results in patients with intrinsic brain tumors when comparing lesions of all lobes.

Studies comparing lesions of the left and right hemisphere found that left hemispheric lesions were associated with the impairment of verbal functions, while right hemispheric lesions led to cognitive disorders of face perseption^21^. Affection of the left hemisphere was also associated with impairment of attention, verbal learning, and language skills^22^. In our study cohort, patients with left frontal lobe lesions achieved lower cognitive test scores for attention, executive functions and memory. Patients with right frontal lobe lesions scored significantly better in most of the subtests for memory. Patients with lesions of the right frontal lobe scored significantly lower TMT-A test results. In accordance with these findings a study dealing with moyamoya disease and reperfusion surgery observed cognitive improvement in patients with increased postoperative right frontal perfusion^23^.

The human parietal lobe is traditionally referred to as the association cortex. It is attributed key functions in processing sensory information including perception, decision making, numerical cognition, integration, speech comprehension, and spatial awareness^24^. In accordance with this, patients with lesions in the parietal lobe had significantly lower test scores in the categories attention and memory and showed a significant impairment in verbal association skills (Wechsler Memory Scale). Those results underline the key role of the parietal cortex as a sensorimotor interface that converts memory and attention tasks derived from visual input into articulatory or motor representations. Other studies found patients with parietal lesions to have more difficulties in force control, guidance of movements, and integrating the image of their own body into the environment^25^.

For patients with frontal lobe lesions, impairment was notable in the Stroop color-word test and the TMT-A test, suggesting a strong influence of the frontal lobe in planning complex actions and novel problem solving. These results are in accordance with previous studies that found frontal lobe damage (e.g., infarcts, abscesses, and tumors) to be associated with increased errors in the Stroop color-word test, slow response times, reduced fluid intelligence and impaired executive functions^26,27^.

Unexpectedly, tumors located in the temporal lobe did not significantly affect results of tests for auditory components. The auditory association cortex has been shown to play a key role in analyzing word meanings and speech signals^28^. Therefore, it is plausible that reactions to single tone-signals, as used in our testing battery, are still possible, while processing complex speech would already be impaired. Patients with temporal lobe tumors showed impairment of memory functions with inferior test results in the VLMT. These results are supported by other studies analyzing different temporal brain lesions^29^.

Previous studies found fluency tasks (category and letter) to be highly sensitive in (low-grade) glioma patients, with significantly impaired test results for patients with lesions of the temporal lobe^30,31^. In this study, no significant correlations were found. Verbal fluency tasks are tailored to assess verbal ability and executive control. For this type of task subjects need to access their mental lexicon in order to retrieve words as well as simultaneously involve executive control processes to meet certain constraints such as avoiding repetition. This reflects the versatility of cognitive processes needed to successfully accomplish those tasks. Therefore, it is not surprising, that word fluency was not related to a specific location in our study population.

Only one of our patients was diagnosed with a tumor of the occipital lobe. Therefore no meaningful statistical evaluation could be deducted. Recent studies analyzing neurological and neuropsychological characteristics of a large number of patients with occipital lobe infarctions found memory and visual disorders (lower test scores in the Rey Osterrieth test, visual-field abnormalities) and difficulties in reading^32^.

Apart from the tumor location, general parameters such as age, tumor volume, KPS scores, and WHO grade had a significant influence in all cognitive domains among our study patients. Since a larger tumor volume obviously compromises a bigger part of the brain and respective lobes, alterations and impairment of the corresponding cognitive functions are to be expected. In previous studies these findings were observed for intrinsic brain tumors as well as for brain metastasis^10,33^.

In addition to the cortical tumor location, subcortical injuries are increasingly recognized as predictors for neurological outcome^34^. Therefore merely attributing the tumor to a certain cortical location or lobe may not fully trace the influence of tumor location to neurocognitive impairment.

WHO grade, or more specifically the growth rate of tumors and its relation to patients’ cognitive functions is currently under discussion^8,10^. An inverse correlation of tumor growth rate and decrease in cognitive function appears plausible. Mechanisms of neural plasticity are considered to differ significantly in slow and fast growing brain lesions. These findings explain our observations, and those of other studies, of reduced cognitive function in more malignant brain tumors independent from lesion volume, age, and tumor location^9,35,36^.

The influence of age, KPS score, and general patient condition on neuropsychological test battery results is not surprising. However, an important finding of our study is that general parameters such as age, KPS score, tumor size, and WHO grade are of higher importance for neurocognitive function than the respective tumor location.

Nowadays, medical treatment is changing from standardized therapy concepts to more individualized regimes. Uniquely tailored health care, individually adjusted to each person’s situation minimizes side effects, improves outcome, and helps preserve life quality. Therefore, knowledge of patient-specific influence factors on the progress and outcome of the disease is essential, especially in the field of cancer treatment. Extensive neurocognitive testing was conducted in this study to assess factors influencing neurocognitive function in patients with intrinsic brain tumors. In comparison to other studies we did not only consider different lobe locations of lesions or the influence of laterality in cohorts with highly pre-selected patients, but rather aimed to systematically assess a large range of risk factors for neurocognitive impairment.

The limitations of this study are: as an exploratory study, we did not correct for multiple testing. Risk factors were assessed and compared among our study patient collective only and not statistically compared to a normative sample. As neurocognitive function is influenced by multiple factors, a meaningful comparison with an adequate normative sample is problematic. Since risk factors were only evaluated in affected patients, we assume that the risk of underestimating influencing factors is higher than to overestimate them. Another limitation is the lack of language tests, as impaired language may influence test results.

## Conclusion

Extensive neurocognitive testing was conducted in this study with precise analytical methods to assess the impact of intrinsic brain tumors on complex brain functions. Comparing results among neuroepithelial tumor patients enables the identification of risk factors for cognitive impairment. Impairment of memory-function and attention were found in patients with lesions of the right hemisphere. Therefore, incorporating memory and attention task into clinical practice for these patients and not only for patients with lesions of the dominant hemisphere might be advisable. According to the present study general parameters such as age, KPS score, tumor size, and WHO grade are equally as important for neurocognitive function as the respective tumor location.

## References

[1] Taphoorn MJ, Klein M (2004). Cognitive deficits in adult patients with brain tumours. Lancet Neurol.

[2] Brown PD (2004). Importance of baseline mini-mental state examination as a prognostic factor for patients with low-grade glioma. Int J Radiat Oncol Biol Phys.

[3] Meyers CA, Hess KR, Yung WK, Levin VA (2000). Cognitive function as a predictor of survival in patients with recurrent malignant glioma. J Clin Oncol.

[4] Prabhu RS (2014). Effect of the addition of chemotherapy to radiotherapy on cognitive function in patients with low-grade glioma: secondary analysis of RTOG 98-02. J Clin Oncol.

[5] Reijneveld JC (2016). Health-related quality of life in patients with high-risk low-grade glioma (EORTC 22033–26033): a randomised, open-label, phase 3 intergroup study. Lancet Oncol.

[6] Talacchi A, Santini B, Savazzi S, Gerosa M (2011). Cognitive effects of tumour and surgical treatment in glioma patients. J Neurooncol.

[7] Mattavelli G (2012). Decision-making abilities in patients with frontal low-grade glioma. J Neurooncol.

[8] Wefel JS, Noll KR, Rao G, Cahill DP (2016). Neurocognitive function varies by IDH1 genetic mutation status in patients with malignant glioma prior to surgical resection. Neuro Oncol.

[9] Klein M (2016). Lesion momentum as explanation for preoperative neurocognitive function in patients with malignant glioma. Neuro Oncol.

[10] Noll KR, Sullaway C, Ziu M, Weinberg JS, Wefel JS (2015). Relationships between tumor grade and neurocognitive functioning in patients with glioma of the left temporal lobe prior to surgical resection. Neuro Oncol.

[11] Satoer D, Visch-Brink E, Dirven C, Vincent A (2016). Glioma surgery in eloquent areas: can we preserve cognition?. Acta Neurochir (Wien).

[12] Cockrell JR, Folstein MF (1988). Mini-Mental State Examination (MMSE). Psychopharmacol Bull.

[13] Zimmermann, P. F. Testbatterie zur Aufmerksamkeitsprüfung (TAP). *Psychologische Testsysteme* (2009).

[14] Wiese W, Kroj G (1972). [Studies on the correlation between intelligence (Wechsler) and the ability to concentrate (Brickenkamp’s testd2)]. Z Exp Angew Psychol.

[15] Tombaugh TN, K J, Rees L (1999). Normative data stratified by age and education for two measures of verbal fluency: FAS and animal naming. Archives of Clinical Neuropsychology.

[16] Russell EW (1982). Factor analysis of the Revised Wechsler Memory Scale tests in a neuropsychological battery. Percept Mot Skills.

[17] Muller H, Hasse-Sander I, Horn R, Helmstaedter C, Elger CE (1997). Rey Auditory-Verbal Learning Test: structure of a modified German version. J Clin Psychol.

[18] Min-Sup Shin S-YP (2006). Se-Ran Park. Clinical and empirical applications of the Rey-Osterrieth Complex Figure Test. nature protocols.

[19] Stroop JR (1935). Studies of intereference in serial verbal reactions. Journal of Experimental Psychology.

[20] Lange, K. W., Aschenbrenner, S. & Tucha, O. Regensburg Word Fluency Task – a new test for the assessment of verbal fluency. *European Archives of Psychiatry and Clinical Neuroscience* **250** (2000).

[21] Scheibel RS, Meyers CA, Levin VA (1996). Cognitive dysfunction following surgery for intracerebral glioma: influence of histopathology, lesion location, and treatment. J Neurooncol.

[22] Noll KR, Ziu M, Weinberg JS, Wefel JS (2016). Neurocognitive functioning in patients with glioma of the left and right temporal lobes. J Neurooncol.

[23] Lei Y (2017). Postoperative executive function in adult moyamoya disease: a preliminary study of its functional anatomy and behavioral correlates. J Neurosurg.

[24] Culham JC, Kanwisher NG (2001). Neuroimaging of cognitive functions in human parietal cortex. Current Opinion in Neurobiology.

[25] Freund HJ (2003). Somatosensory and motor disturbances in patients with parietal lobe lesions. Advances in neurology.

[26] Stuss DT, Floden D, Alexander MP, Levine B, Katz D (2001). Stroop performance in focal lesion patients: dissociation of processes and frontal lobe lesion location. Neuropsychologia.

[27] Roca, M. *et al*. Executive function and fluid intelligence after frontal lobe lesions. *Brain*, 10.1093/brain/awp269 (2009). 10.1093/brain/awp269 PMC2801324 19903732

[28] Sharp DJ, Scott SK, Wise RJ (2004). Retrieving meaning after temporal lobe infarction: the role of the basal language area. Annals of neurology.

[29] Esfahani-Bayerl N (2016). Visuo-spatial memory deficits following medial temporal lobe damage: A comparison of three patient groups. Neuropsychologia.

[30] Banerjee P (2015). Association between lesion location and language function in adult glioma using voxel-based lesion-symptom mapping. NeuroImage: Clinical.

[31] Campanella F, Mondani M, Skrap M, Shallice T (2009). Semantic access dysphasia resulting from left temporal lobe tumours. Brain.

[32] Kraft A (2014). Neurological and neuropsychological characteristics of occipital, occipito-temporal and occipito-parietal infarction. Cortex; a journal devoted to the study of the nervous system and behavior.

[33] Habets EJJ (2016). Neurocognitive functioning and health-related quality of life in patients treated with stereotactic radiotherapy for brain metastases: a prospective study. Neuro-oncology.

[34] Trinh VT (2013). Subcortical injury is an independent predictor of worsening neurological deficits following awake craniotomy procedures. Neurosurgery.

[35] Desmurget M, Bonnetblanc F, Duffau H (2007). Contrasting acute and slow-growing lesions: a new door to brain plasticity. Brain.

[36] Kesler, S. R., Noll, K., Cahill, D. P., Rao, G. & Wefel, J. S. The effect of IDH1 mutation on the structural connectome in malignant astrocytoma. *J Neurooncol*, 10.1007/s11060-016-2328-1 (2016). 10.1007/s11060-016-2328-1 PMC5377918 27848136

