# Publisher Correction: Factors influencing neurocognitive function in patients with neuroepithelial tumors

**DOI:** 10.1038/s41598-018-21908-7

**Published:** 2018-03-06

**Authors:** Jens Gempt, Nicole Lange, Stefanie Bette, Sarah Charlotte Foreman, Jasmin Hernandez Cammardella, Jennifer Albertshauser, Corinna Gradtke, Niels Buchmann, Yu-Mi Ryang, Friederike Schmidt-Graf, Bernhard Meyer, Florian Ringel

**Affiliations:** 1 Neurochirurgische Klinik und Poliklinik, Klinikum rechts der Isar, Technische Universität München, Ismaninger Str. 22, 81675 München, Germany; 2 Abteilung für Neuroradiologie, Klinikum rechts der Isar, Technische Universität München, Ismaninger Str. 22, 81675 München, Germany; 3 Neurologische Klinik und Poliklinik, Klinikum rechts der Isar, Technische Universität München, Ismaninger Str. 22, 81675 München, Germany

Correction to: *Scientific Reports* 10.1038/s41598-017-17833-w, published online 19 December 2017

In the original version of this Article, the author Sarah Charlotte Foreman was incorrectly indexed. This error has now been corrected.

